# Mucosal histopathology in celiac disease: a rebuttal of Oberhuber’s sub-division of Marsh III 

**Published:** 2015

**Authors:** Michael N Marsh, Matt W Johnson, Kamran Rostami

**Affiliations:** 1 *Department of Gastroenterology, Luton & Dunstable University Hospitals Trust, Luton, United Kingdom*; 2 *Wolfson College, University of Oxford, United Kingdom*; 3 *Department of Gastroenterology, Alexandra Hospital, Worcestershire, United Kingdom*

**Keywords:** Scanning EM, Marsh celiac classification, Marsh III lesion, Mosaic, Mucosal surface contour

## Abstract

Individuals with particular genetic backgrounds develop immune responses to wheat proteins and become ‘gluten-sensitised’. Mucosal pathology arises through activated mucosal T lymphocytes, resulting in a graded, adverse reaction between particular genes and wheat proteins. Given these varied influences, the Marsh Classification broadly itemises those stages through which a normal mucosa (Marsh 0) evolves in becoming ‘flat’ (Marsh I, II, III).

Recently, Oberhuber and colleagues suggested that Marsh III lesions required subdividing into a, b, c categories. We critically examined these subdivisions by means of correlative light and scanning electron microscopy (SEM). Our results demonstrate that Oberhuber’s classification is untenable. In our view deriving from our observations, the artificial subdivisions proposed by those authors actually reflect misinterpretations of the true architectural contours of flat mucosae. Although these workers refer to “villous projections”, SEM demonstrates that no such structures are present on flat - or immediately recovering – mucosae.

Our data revealed on the surfaces of flat (Marsh III) mucosae, large open “basins”, surrounded by raised collars - the latter, when viewed in histological section, being easily misconstrued as “villi”. It seems that with subsequent upward growth, these collars coalesce into low ridges, thence becoming broader and higher convolutions. It is noticeable that there are more open spaces on the surfaces of flat mucosae than was appreciated hitherto. We conclude that Oberhuber’s revisions of Marsh III into three subcategories (a, b, c), are misinterpretations of the histological appearances of flattened mucosae. Therefore, histopathologists when classifying celiac mucosae, since they add nothing either of diagnostic, nor prognostic, value should resist these subcategories.

## Introduction

The mucosal response to wheat protein ingestion in genetically predisposed subjects has, over the recent years been shown to be increasingly complicated. It is no longer valid to define “celiac disease” solely in terms of a severely damaged, flat with complete effacement of its villi. However, that was the accepted position until 1992 when one of us established a classification identifying the prominent immuno-histopathological phases through which the mucosa passes in becoming flat (1).

 That was an inevitable development, in the face of certain isolated case-reports hinting at the possibility of progressive changes over time (2- 4) but which, hitherto, had never been brought into a refined definitional mode. The origin of that classification depended on two factors: 

 First that a flat mucosa does not arise *per se*: it has to evolve. Previous to the publication of the Marsh Classification (1), no one had apparently asked how a flat mucosa comes about. It was assumed, from the first descriptions of the severe lesion (5, 6), that this was the sole tissue response to gluten ingestion. That position survived for over 40 years.

 Second, and more importantly, it became apparent that the interplay between the sensitising grass-derived proteins of wheat, barley and rye (‘prolamins, avenins and secalins’), and the relevant genetic background, initiates mucosal damage through activation of intestinal CD4+ TCR + T-cells within the lamina propria (7).

 Logically, then, the interaction between the relevant genes and gluten ingestion must occur *before *mucosal damage has begun. In other words, no individual destined to become gluten-sensitised, is born with an abnormal mucosa. The damage incurred is clearly secondary to immunological processes triggered within the mucosa, and exacerbated by additionally recruited inflammatory mechanisms contributing to cell loss, tissue damage and mucosal remodelling (8). The progressive alterations to mucosal architecture, related analogically to other immunopathological changes seen in tropical sprue, giardiasis, childhood protein hypersensitivities, and the graft-versus-host reaction, underpinned the thinking behind the Marsh Classification. Importantly, and for those very reasons, the classification included an initial Type 0, or histologically ‘normal-looking’ mucosa, a view seemingly backed up by several recent studies (9-12).

 In a subsequent re-evaluation of the Marsh Classification, Oberhuber and colleagues (13) suggested that the severest Marsh III stage required alteration into three subcategories (a, b, c). The observations reported in this paper re-examine those modifications, and show them to be based on uncontrolled interpretations of the Marsh III lesion. 

## Correlative observations with optical/scanning electron microscopy

**Figure 1:**
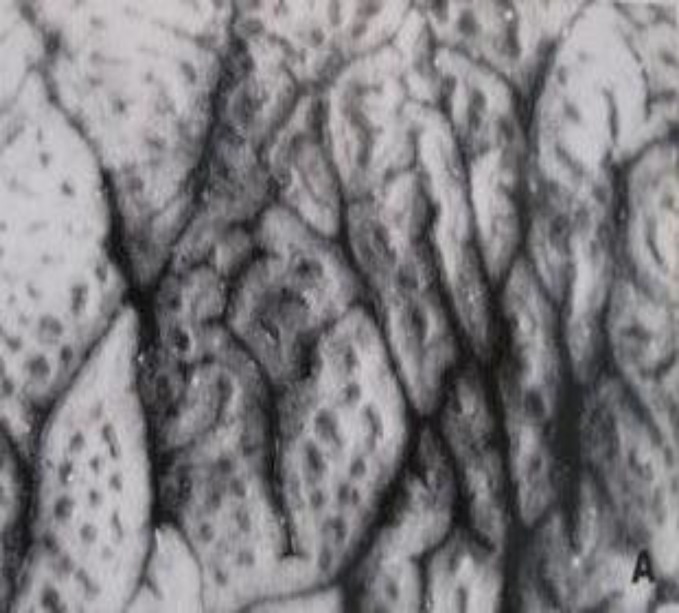
Dissecting microscope appearances of a flat ‘mosaic’ specimen. The surface is broken by irregular plateaux and bounded by deep furrows. The depressions on the surfaces of the plateaux, however, are not the openings of individual crypt tubes.

 The surface of a flat lesion is not devoid of structural detail. When observed through the dissecting microscope, the surface of a severe (Marsh III) celiac lesion is perforated by several openings (Fig 1), which formerly were thought to be the openings of individual crypts. 

**Figure 2:**
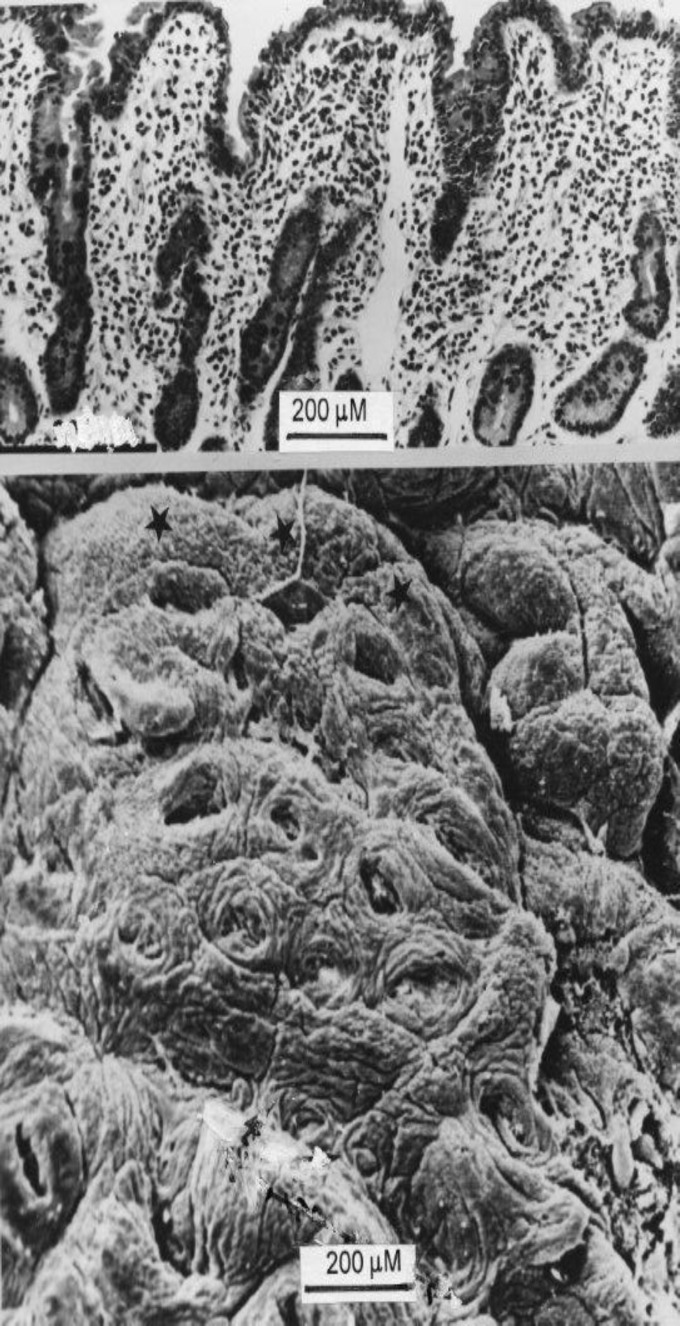
The SEM reveals that the surface openings (see Fig 1) are large crevices up to 200mM in length. Within the depths of some of these crevices, the openings of individual crypt tubes are visible. Each crevice (basin) is surrounded by concentric arrays of enterocytes (“collars”). Some collars are elevated above the plane of flattening.

**Figure 3:**
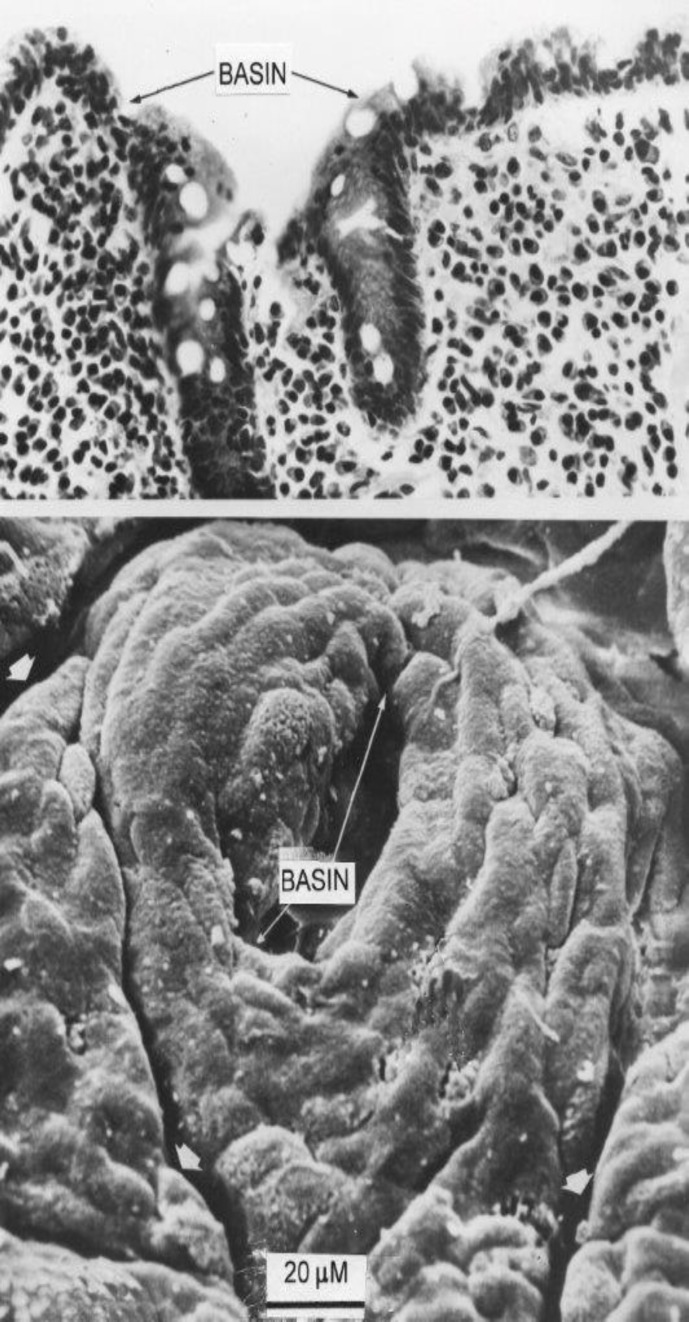
Close-up of another individual basin and surrounding crypt collars. Above, a corresponding histological view of the same specimen, showing two individual crypt tubes opening into the basin. On left, the section through the collar could be mistaken for a “villous” projection.

With the SEM, flat mucosae demonstrate three characteristic features (Fig 2): (a) extremely big surface perforations, (b) concentric rings of enterocytes bordering these openings, which (c) may be raised to variable degree. This feature is illustrated in close-up (Fig 3); the histological section shows a glancing section of one such basin, with the left hand collar suggesting a projecting “villus”. Since we know that the rate of loss of surface enterocytes from a severe lesion is roughly increased six fold (14) compared with normal mucosae, we could infer that these collars represent large numbers of cells being produced by, and recently emigrated from, the highly proliferative regions of many crypts. 

**Figure 4:**
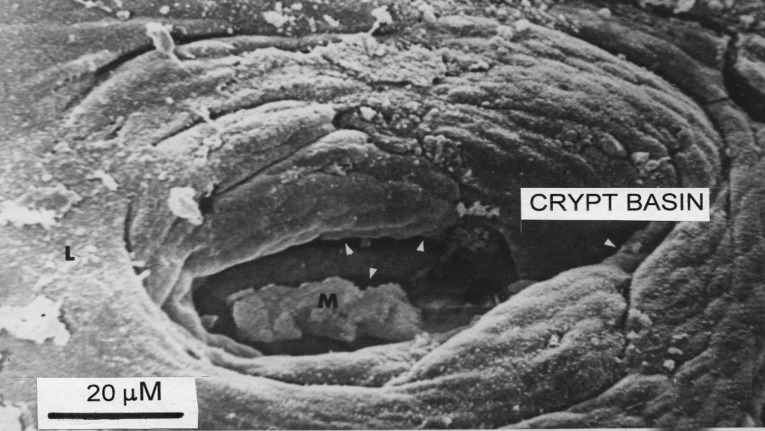
Closer view of a single crypt basin. White arrow heads locate openings of individual crypt tubes at bottom of basin. M, mucus blob.

 Second, we wish to emphasise that the large openings on the surface of flat mucosae are not individual crypt tubes, as originally thought, but substantial “basin-like” cavities (15), ranging up to 200µM in width and in depth. With the increased depth of field-focus of the scanning microscope, it is evident that several individual crypts open into the lower regions of these large, oval basin-like orifices (Fig 3, 4). It should be noted; contrary to Oberhuber, that there are no “atrophied” remains of villi on the surfaces of these severely damaged mucosae.

**Figure 5:**
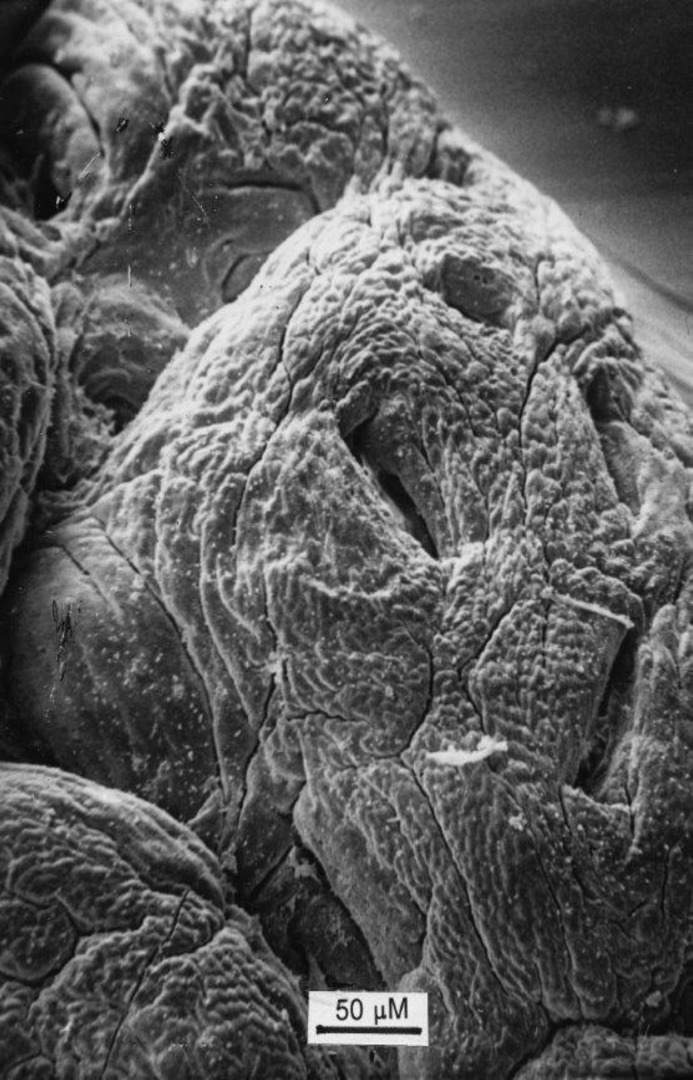
SEM appearances of a small mosaic plateau bearing the large openings of several basins. Some may be seen to be perforated in their depths by individual crypt tubes. Other openings of individual crypt tubes are visible at the base of the adjacent furrow to the left of the plateau illustrated.

Another reason why we suggest that Oberhuber and colleagues were incorrect lies in their failure to take account of the mosaic structuring of the mucosal surface which comprises furrows lying between very large, elevated and irregularly-shaped plateaux (Fig 1, 5). The basis of the mosaic appearance is an important surface mucosal feature, which has never been explained, and largely forgotten about during recent years. The surfaces of these curious plateaux are variably *raised* to around ~200µM above the openings of individual crypts, the latter openings also occupying the furrows between the edges of these lozenge-shaped mucosal elevations (Fig 5). 

That the surfaces of these plateaux lie significantly above the linear series of individual crypt tubes (opening directly into the furrows separating adjacent mosaic plateaux) has considerable significance in terms of the mechanisms causing villous effacement. This feature is dealt with in our discussion below, our argument being that villus effacement is not an “atrophic” process, and that “atrophic” remnants of villi are not scattered across the surfaces of severely flattened (Marsh III) mucosae.

 When the sequence in mucosal flattening was first recognised (1), it was realised that there was no available intermediary stage between the Marsh II and III lesions. Indeed, no such intermediary stage has ever been recognised and neither do we know how long it takes. Indeed, this elusive II-III transition may be completed very quickly, so that its appearance in biopsies is less frequently encountered. However, as an alternative in trying to bridge this gap, and observe what might be happening at this important stage in mucosal flattening, we decided to observe the mucosa during its early responses to gluten restriction.

**Figure 6:**
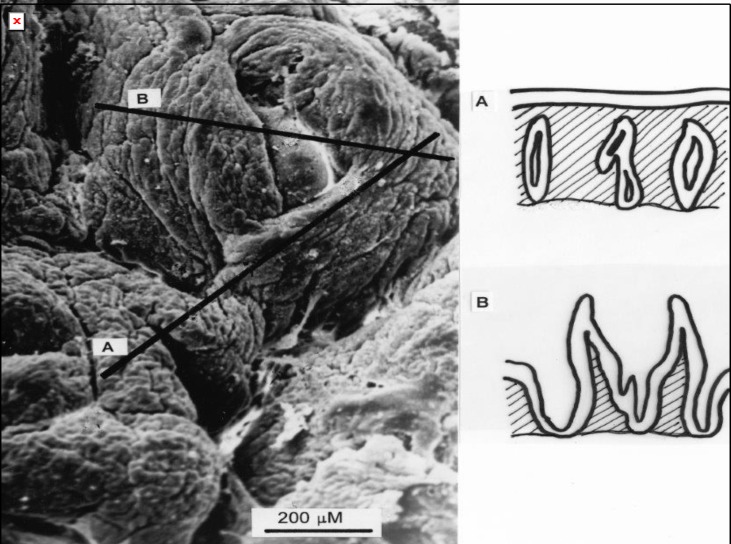
This specimen, obtained after 5 months of dietary gluten restriction, reveals two elevated, adjacent collars that appear to have joined to form a curvilinear ridge. These ridges will ultimately thicken to form low ‘convolutions’ from which true villous projections will ultimately take origin, although at a much later stage in mucosal regeneration. On the right, imaginary appearances of random sections across this specimen are shown. One (A) reveals the result of a section along the flat surface contour relevant to the conjoined ridge. The other (B), passing through the collared basin, reveals pseudo-villous contours, even though overall, the specimen is still flat. This is a prime example of how the surface contour varies within a micro-region across every specimen, and clearly demonstrating that any random, thin histological section cannot provide useful information about surface shape and contour.

 A representative view (Fig 6) is from a specimen obtained from a patient who had recently been started on gluten restriction: this specimen is still flat. It reveals considerably elevated concentric rings of cells forming collars, but also their cohesion along the surface of the mucosa. 

Presumably, in the absence of dietary gluten, these emerging cells are now able to survive for a longer period on the mucosal surface thus to contribute to the regenerative process. However, despite its flatness, vertical sections through this area could both produce differing appearances, dependent on where a random plane of sectioning happened to pass. For example, through one plane the mucosa would look entirely flat, while with another section-orientation and slight difference in sectioning angle, a view from a section taken across these elevated collars would appear to be of a lesser degree of flattening, and even suggest the presence of “villi”. However, no such conclusion is, in fact, possible, as evident from the SEM appearances. 

 As we have stressed, in regard to the earliest phases in mucosal regeneration, attention has to be focussed on the crypt collars. These not only become more prominent, but also taller with time. Despite the dietary control, the surface characteristics of the mucosa change very slowly. However, with progressive regeneration, it would appear that adjacent collars fuse with each other, forming low ridges, which after further enlargement, become, as seen through the dissecting microscope, higher and wider convolutions. However, those final stages in mucosal regeneration are not our present concern.

 The biopsy pictures shown here reveal how easy it is for sectioned projections (crypt collars or early convolutions) from the epithelial surface to be misinterpreted as “partial” or “completely “atrophic villi, when no such structures actually exist. It is necessary to control such interpretations of isolated sections with parallel, panoramic views taken by SEM of the same specimen, as these figure plates (Figs. 2-6) so pointedly demonstrate. These observations reveal how random sections through the mucosa (as invariably used in routine histopathological assessments of biopsy specimens) bear scanty relationship to the broader mucosal contours as demonstrated by SEM.

## Discussion

 Our study brings to light structural features of so-called “flat” (Marsh Type III) celiac lesion as observed by SEM. These features appear not to be widely appreciated although they considerably temper interpretations of the mucosal surface, especially if based solely on inspection of one or two histological sections alone.

 Together, our results demonstrate that: (i) there are no “villi” associated with any grade of a flattened mucosa (ii) there are basin-like openings (or wells) up to 200 µM or more in width into which several individual crypt tubes open, and whose periphery is associated with concentric rings of surface enterocytes (“collars”) (iii) basins seem to be present on the surface of the large mosaic plateaux which comprise the surface of some mucosal specimens and whose presence requires explanation, (iv) misinterpretations of so-called “villi” are, in fact, cross-sections through raised crypt “collars” on the mucosal surface and (v) the (re)-formation of villi is, in fact, a very late occurrence in the regenerative process, deriving from convolutions which, themselves, derive from the progressive merging of elevated crypt collars. The progressive elevation and enlargement of crypt collars presumably progresses as the surface epithelium stabilises, following dietary gluten withdrawal.

 On these grounds, the misinterpretations occasioned by Oberhuber’s team in their attempt to re-classify the Marsh III lesion into three new, discernible levels: a, b, and c, become apparent. That new sub-classification is impossible to substantiate on the basis of our demonstration of surface basins, crypt collars and their progressive enlargement as the epithelium stabilises, once gluten restriction is imposed and the cells no longer are desquamated in high numbers from the mucosal surface. 

 Further problems with Oberhuber’s paper (13) rest on technical criticisms. These criticisms are necessary, since the paper itself attempts to put forward a new position. Firstly, these authors merely reproduce Marsh Types 0, I, and II lesions, unsupported by any new, empirical data. Second, they pay scant regard to the IIIa, b, c classifications originally put forward by Rostami and others (16) and which must have been available to Oberhuber’s team before they went to press. Thirdly, the minute specimens published in their paper (their Figs 4, 5), which attempt to underpin their attempts to reclassify Marsh Type III lesions, could hardly be deemed acceptable. In both these figures, the specimens are twisted and sometimes sectioned almost horizontally to the mucosal surface. Therefore, it is impossible to assert (from their Fig 3, for example), that ‘the villi show a mild atrophy’ for two important reasons.

 First, no independent proof is offered to confirm that these are “villi”. More importantly, no definition of “atrophy” is given. The description offered for their Fig 5 is that ‘the “villi” are only very short (“marked villous atrophy”), sometimes appearing tent-like’. However, the specimen offered as proof was not sectioned precisely in the vertical plane. 

 Second, the so-called “villi” are hardly any shorter or longer than those purported to be discernible in Fig 4, and it not clear what “tent-like” means. Again, the meaning of “marked villous atrophy”, and its differentiation from only “mild atrophy” is not defined, and hence in our view, hardly credible. 

 Third, what are their specific criteria for defining the end product as “atrophic”? Similar objections apply to the more recent paper by Dickson and colleagues (17) whose interpretations of “villous” flattening are inconsistent with the categories proposed by Oberhuber. Indeed, when the illustrated samples provided by each set of authors are compared, side-by-side, the vast discrepancies in interpretation becomes apparent, indicative of the uncontrolled and obviously arbitrary perceptions of Marsh IIIa, b, c which exist among various authors. Furthermore, these discrepancies suggest that further reclassifications of the original Marsh scheme, embodying the Oberhuber changes (18,19) can be seen as unacceptable, for the reasons already stated.

 The structure of the jejunal mucosa is extremely complex, a complexity not evident when only thin, individual sections of the mucosa are observed in isolation. There is one study known to us in which that complex structure has been realised, through the construction of wax models of mucosae obtained from three, apparently normal human subjects (15). Those wax reconstructions revealed the enormously varied micro-anatomy at, and around, the villous-crypt interface, where up to twenty crypts might encompass one single villus. Without that insight, it would be impossible to perceive the almost unimaginable complexity of this critical junctional zone from histology alone.

 Moreover, crypts were shown by these models (15) to open into large, common ‘circumvillar basins’, ~100-200µM deep. This kind of structural arrangement could never be inferred from single sections alone. It might be possible, perhaps, to discover such a system of crypts if sequential (vertical) serial sections through a tissue slab were carefully examined. Horizontal sections also reveal this pattern (17) although Loehry and Creamer (see their Fig 3a,b) may not have fully appreciated what they were demonstrating. In passing, it should be noted that these basins are open spaces. That is, their “structure” arises from the configurations of adjacent tissues: it is extremely difficult to envision large spaces in histological sections. For example, it is more than likely that the left-hand portion of Fig 3 in Dickson’s paper (17) probably represents a section through a basin, although the authors erroneously interpret both sides of the raised collars surrounding this basin as “degenerate villi”. There is no entity (known to us) definable as a “degenerate villus”.

 A further critical aspect of the mucosa is raised by the question why the mucosal surface is divided into large, irregularly-shaped plateaux, and what possible bearing these could have in terms of villous effacement. It is our view that the mosaic is related to the process (es) of villous loss. Therefore, we suggest, occurs by two concurrent processes – (a) progressive reduction in the height and in the widening of villi, but accompanied (b) by the upward growth of the inter-villous ridges identified by Loehry & Creamer (20). Thus, as a result of these two processes, many adjacent villi become conglomerated into broad, irregularly-shaped plateax. 

 Further evidence for this view was provided by Padykula (21) in studies of the histochemical characteristics of the epithelium of flattened mucosae. She and her colleagues demonstrated that the upper regions of the elongated crypts of flat mucosae contained enterocytes (their “zone 2”) bearing the tinctorial qualities of villous cells expressing certain enzymes, such as esterase and alkaline phosphatase. It is our view that these particular cells correspond to the cells lining the basins demonstrated by SEM, and in the reconstructed wax basins of Cocco already mentioned (15). Padykula’s study (21) thus indicates that the surface of a flat (coeliac) mucosa does not normally lie at the crypt-villus interzone, (as if the villi had been simply shaved off at the tops of individual crypts), but is *raised up *to a maximum height of approximately 200µM *above* *that level*, corresponding to the variable height of the mosaic plateaux. That is, mosaic plateaux, comprising conglomerations of many villi, represent villous territory, and not that of crypts. Analysing upper mucosal cells for the RNA gene transcripts underlying synthesis of these digestive enzymes could reconfirm such a view. Moreover, such data would provide incisive criteria for the contested decision for distinguishing precisely where crypts terminate, and villous territory begins, a problem, which could put in jeopardy the recent analysis based on villous heights and crypt depths by Taavela and colleagues (22). Nevertheless, the thrust of this argument firmly provides another critical reason against believing that the assertions of Oberhuber et al. are tenable (13). 

 In this respect, we should also inspect recent proposals that cytotoxic killing of enterocytes results in villous flattening (23, 24). Such conclusions do not cover the full picture, and demand a far more expansive and informed approach, inclusive of all other factors operative in the process. The reason for seriously questioning that viewpoint is that if the enterocytes are stripped from the mucosa, the villous cores, including subepithelial basement membrane and the vascular elements of arterioles, capillaries and venules, remain unscathed (20). Simply removing enterocytes from the mucosa does not result in mucosal (even typically celiac-type) flattening. Indeed, the death of surface enterocytes, alone, does not provide a cast-iron reason for mucosal flattening. Moreover, we must remember that these workers were only working with already flat (Marsh III) mucosae. The critical question is: are such processes already operative with early Marsh 0 and Marsh I lesional pathology? If not, then we certainly need to think again.

 Clearly, many other processes involving enzymatic dissolution of matrix structures by metalloproteins (25, 26) play important roles in the concurrent remodelling of the mucosa which, together with villous effacement and emergence of inter-villous ridges, entails the intervening stage of mosaic plateaux formation. Since flattening and progressive mucosal remodelling begin with the “normal” mucosa (Marsh 0), it is neither logical nor convincing to suggest that flattening occurs solely with, or is initiated by, immunological processes identifiable only within severe Marsh III lesions.

 The variable depth of the surface basins suggests that the thickness of the raised “mosaic slabs” is dependent on the degree to which the effacement process has progressed. From that it would follow that the thinnest mucosae (in contrast to the more luxuriant, thicker specimens) are those in which the processes of effacement have now removed all trace of the mosaic superstructure. It may be these ‘end-stage’ specimens that are becoming non-regenerative and therefore, in this extreme, truly “atrophic” (with or without some form of lymphomatous process in progress) – the original Marsh IV category. But here again, we have no idea whatsoever as to the mechanisms, which prevent mucosal regeneration taking, place, and how these processes impinge on a failure of crypts to deliver new regenerative cells. This, therefore, calls for a completely renewed initiative in determining why some crypts regenerate, while others fail.

 Next, it is necessary to account for the marked *expansion* of lamina propria volumes, revealed by computerised morphometry, as flattening progresses (27). Part of that increase may subsume the growth *upwards* of inter-villous ridges in the formation of convolutions demonstrated by Loehry (20), but would also include tissue oedema and cellular infiltrations. These considerations point to the necessity of understanding the molecular cross-talk between epithelial and connective tissue elements in the remodelling process. Clearly the remodelling of the micro-vasculature is of critical importance, but we know little of the dynamics of this process. Further work is needed to elucidate the cell biology here, and to fully understand how the immunological processes involved are co-ordinated in this process.

 All these proposals are testable, and ideally would involve a systematic study of the genes recruited during the course of effacement, including those concerned with dissolution and reconfiguring of the lamina propria (and progressively, through each of the stages Marsh 0-III, IV). They might possibly lead to newer, stringent diagnostic procedures as knowledge of the earliest phases involved in initiating the involution of villi are set in motion. What is clear, however, is that the mosaic slabs should be regarded as an amalgamation of villous epithelial cells together with their associated underlying connective tissue matrix, including a remodelled microvasculature. On those grounds alone, there could be no possibility of an atrophic process applied to single villi, as implied in the paper of Oberhuber and colleagues.

 Despite these considerations, our message is clear. Oberhuber’s modifications to the original Marsh classification have been shown, in this study, to be grounded on an incorrect interpretation of the crypt-villus interzone, and in particular, to a failure to appreciate the presence of crypt collars and their subsequent amalgamation into ridges and thence to higher, fatter convolutions. For this kind of analysis, it is evident that histological observations require independent control through a monitoring of surface ultrastructure. Only in that way can the information obtained from thin vertical sections be fully understood, in relation both to the surface ultrastructure of any flat mucosa, and how the varied structures on the surface progressively change as regeneration occurs.

 Since regeneration of flat mucosae demonstrably occurs, the continued use of “atrophy” terminology (6) is likewise outmoded and unhelpful. In our opinion, it would be necessary to offer far more robust, ancillary evidence, accompanied by a greater demonstration of technical excellence, and independent verification, before the changes proposed could be universally acceptable. 

 We strongly disagree with the conclusions of Oberhuber and colleagues and do not believe that they offer a sound basis *‘for a standardised report scheme for pathologists’* – or for anyone else, for that matter. It is surprising that their conclusions have been followed and adopted so widely without logical and critical evaluation.
